# Aerobic and strength exercises for youngsters aged 12 to 15: what do parents think?

**DOI:** 10.1186/s12889-015-2328-7

**Published:** 2015-09-30

**Authors:** Gill A. ten Hoor, Ester F. C. Sleddens, Stef P. J. Kremers, Annemie M. W. J. Schols, Gerjo Kok, Guy Plasqui

**Affiliations:** Department of Human Biology, Nutrition and Translational Research in Metabolism, Maastricht University Medical Center+, P.O. Box 616, 6200 MD Maastricht, The Netherlands; Department of Work and Social Psychology, Maastricht University, P.O. Box 616, 6200 MD Maastricht, The Netherlands; Department of Health Promotion, Nutrition and Translational Research in Metabolism, Maastricht University Medical Center+, P.O. Box 616, 6200 MD Maastricht, The Netherlands; Department of Respiratory Medicine, Nutrition and Translational Research in Metabolism, Maastricht University Medical Center+, P.O. Box 616, 6200 MD Maastricht, The Netherlands

**Keywords:** Resistance exercise, Adolescents, Attitude, Parenting, Physical activity, Intervention development

## Abstract

**Background:**

Although strength exercises evidently have both physiological and psychological health benefits across all ages, they are erroneously considered to adversely affect health status in youngsters. The aim of this study was to examine parental attitudes towards their child’s physical activity in general, as well as aerobic and strength exercises in particular.

**Methods:**

In total, 314 parents from an online panel representative of the Dutch population completed an online survey about their own physical activity and that of their child (12–15 years old). The study also explored reasons for non-participation, and attitudes about the parents’ own and their child’s physical activity level.

**Results:**

Parents consistently reported a positive attitude towards aerobic exercises, but a less positive attitude regarding strength exercises. Parents were more likely to indicate that their child was not allowed to participate in strength exercises (29.6 %) than aerobic exercises (4.0 %). They thought that strength exercises could interfere with optimal physical development.

**Conclusions:**

This study consistently shows that parents have a positive attitude towards aerobic exercises, but a less positive attitude regarding strength exercises. We suggest testing interventions to increase parental understanding of the advantages of and possibilities for (e.g., facilities) strength training on their child’s health.

**Electronic supplementary material:**

The online version of this article (doi:10.1186/s12889-015-2328-7) contains supplementary material, which is available to authorized users.

## Background

Strength exercises have been shown to contribute to the prevention and reduction of obesity-related health problems (Lloyd et al., 2014). Strength exercises are defined as “exercises whereby an individual is working against a wide range of resistive loads to enhance health” (Lloyd et al., 2014, p1.) They have been shown to improve body composition [1], decrease risk of developing chronic metabolic diseases [2], and contribute to injury prevention (Lloyd et al., 2014). Psychological benefits have also been reported, e.g., improvements in self-concept [3, 4], confidence [5], mood [6], and quality of life [7, 8].

The idea that strength exercises for children and youngsters are detrimental for their health is outdated [6, 9, 10]. In the 1970s and 1980s, it was suggested that strength exercises are harmful for youngsters, particularly during growth, but there is compelling evidence that this is a persistent misperception, lacking in evidence ([9–13], [6]). In fact, more recently, the idea that strength exercises can be beneficial for youngsters (here defined as 12–15 year olds) is being embraced more and more [14–17].

We suggest that an important factor that may contribute to the relatively low participation of youngsters in strength exercises and strength-oriented sports is parental attitudes regarding these types of exercises. Parents play a crucial role in the physical activity-related behaviour of their children [18, 19]. They are largely responsible for the type and amount of physical activity that their child carries out, and are role models who influence their child’s physical activity behaviour [20, 21]. To develop and implement tailored physical activity interventions for youngsters it is important to identify parental attitudes about their children’s participation in such exercises. We suggest that an important factor that may contribute to the relatively low participation of youngsters in strength exercises and strength-oriented sports is parental attitudes regarding these types of exercises. In this study, we investigated parental attitudes towards their own -- as well as their child’s -- physical activities, including strength and aerobic exercises.

## Method

Following pleas for full disclosure [22, 23], all research materials, data, analyses and output are available in a combined .rar archive that can be found in Additional file 1. This study was approved by the Research Ethics Board of the Faculty of Psychology and Neuroscience, Maastricht University, the Netherlands.

### Participants

In order to include a minimum of 300 parents, 600 parents of 12–15 year olds were randomly invited via Flycatcher to participate in this study (Flycatcher has a normal response rate of approximately 50 %). Flycatcher, an online panel representative of the Dutch population (http://www.flycatcher.eu/; ISO 26362 and ISO20252; Dutch quality label, certifying that the panel can be used for social-scientific research), has 1300 registered parents of youngsters aged 12–15 years. After dropout (wrong e-mail address, *n* = 10; bad response, straight lining, or consistent patterns in answering; nonsense answers on open questions (e.g., typing random letters), *n* = 23), 314 parents completed the study (53.2 % response rate).

### Procedure and measures

All participants provided informed consent prior to data collection. The questionnaire consisted of four parts: two parts about the parents themselves, and two parts about the children. First, general questions were asked about the parent’s own physical activity behaviour and their self-reported height (in centimeters) and weight (in kilograms). Subsequently, their attitudes with regard to engaging in physical activity were measured. In the third and fourth part of the questionnaire, similar questions were asked, but then in relation to their child’s physical activity behaviour and attitudes (we measured parental perceptions of their child´s activity and attitudes). Before answering the questions about their child, participants were instructed that all questions were about their youngest child in the age range 12 to 15 years. Questions were formulated by following the guidelines provided by Fishbein and Ajzen [24].

Information about gender, age, and highest level of completed education (categorised into low – none, or primary education; medium – intermediate/high general secondary education or intermediate vocational education; high – college degree or higher) had already been collected by, and was available from Flycatcher. All items were rated on a 7-point Likert scale ranging from 1 (*completely disagree*) to 7 (*completely agree*), unless otherwise stated. Scores on items that measured the same construct were averaged into one scale where internal consistency was sufficient (α > .60; [25]). Scores were recoded such that a higher score reflected a higher or more positive value of the construct in question.

#### Physical activity behaviour

The parents were asked whether they themselves are physically active, what kind of exercise(s) they perform, how often they engage in this type of exercise, the average amount of hours they engage in the exercise(s) per session, and their estimation of the type of exercise they engage in: 1- *aerobic*; 2- *a combination of aerobic and strength, mostly aerobic*; 3- *an equal combination of aerobic and strength*; 4- *a combination of aerobic and strength, mostly strength*; 5- *strength*. If the parent indicated that he or she did not engage in physical activity, we asked what the most important reason(s) for this was (were).

Next, questions about their child’s physical activity behaviour were asked (these were similar to the parent’s own general questions). Additionally, two questions were asked about whether their child is allowed to participate in physical activities with an emphasis on aerobic activities, and whether their child is allowed to participate in physical activities with an emphasis on strength exercises. For both questions the response scale ranged from 1 (*absolutely not*) to 7 (*absolutely*). When the participant answered one of these questions with a score indicating that their child is not allowed to participate in either one of these types of exercises (score <5), we asked what the most important reason(s) for that decision was (were). Due to a malfunction of the online questionnaire, not all parents answered the question, “Is your child allowed to participate in exercises with an emphasis on aerobic components?” (66 missing values). For subsequent linear regressions, these were imputed by a random number based on the same mean score and standard deviation.

#### Parental attitudes about their own physical activity behaviour

Parental attitudes about physical activity in general (5 items), strength exercises (5 items) and aerobic exercises (5 items) were assessed using the general attitude questions proposed by Ajzen and Fishbein (2010; further referred to as ‘general attitudes’). All questions were rated on a 7-point Likert scale, i.e., *“I think my engagement in physical activity/strength exercises/aerobic exercises is very good – very bad; very important – very unimportant; absolutely not necessary – absolutely necessary; very unpleasant – very pleasant, very harmful – very harmless.* Cronbach’s alphas for the scales ranged from .78 to .94.

#### Parental attitudes about their child’s physical activity behaviour

In order to assess (general) parental attitudes about their child’s behaviour, similar questions were asked as those regarding their own behaviour. For items relating to their child, all questions started with, *“When my child participates in physical activity/strength exercises/aerobic exercises, or wants to participate, I think that is…”* Subsequently, more specific parental attitudes were assessed regarding their child’s perceived abilities in terms of participating in the different types of exercises, whether they would allow and encourage exercise in their children, and norms and expectations (further referred to as specific attitudes). The exact questions can be found in Table 1.

**Table 1:** Specific questions about parental attitudes towards their child

Abbreviation	Specific attitudinal questions^a^
Possible	In my current situation, it’s absolutely possible to let my child participate in aerobic/strength exercises
Facilities	There are enough facilities to let my child participate in aerobic/strength exercises
Fit/strong	My child is very fit/strong and healthy, and therefore my child does not have to participate in aerobic/strength exercises.
Worse/better	My child is (1) much worse – (7) much better at aerobic/strength exercises compared to other children of the same age and gender.
Enjoyable	Compared to other children of the same age and gender, my child thinks aerobic/strength exercises are (1) less enjoyable – (7) more enjoyable
Good	My child is good in aerobic/strength exercises
Allowed when wanted	When my child wants to, he/she is allowed to participate in aerobic/strength exercises.
Encouraged when wanted	When my child wants to participate in aerobic/strength exercises, I will encourage him/her.
Expectation	I expect my child to start participating in aerobic/strength exercises

*Note.* ^a^All questions were asked about aerobic and strength exercises separately

#### Child and parental background factors

Various parental background factors were assessed. These included gender, age, and educational level (low - medium - high). Parents were asked to indicate their own weight and height, which was used to calculate parental Body Mass Index (BMI; in kg/m^2^). For descriptive purposes, BMI values were categorised as underweight (BMI < 18.5), normal-weight (BMI ≥ 18.5 and BMI < 25), overweight (BMI ≥ 25), or obese (BMI ≥ 30). Parents were also asked to report their child’s weight and height in order to calculate the child’s BMI. The child’s BMI was then recoded into age and gender-specific BMI z-scores and compared to the national reference population [26]. A child’s BMI z-score > 85^th^ percentile was considered to indicate overweight, and a child’s BMI z-score > 95^th^ percentile was considered to indicate obesity [27]. BMI z-scores < −5 or > 5 were considered unrealistic and were not used for further analyses, as advised by the World Health Organization [28].

### Data analyses

IBM SPSS statistics 20 was used to analyse the data. Descriptive analyses - frequencies (*N*), means (*M*) and standard deviations (*SD*) – were calculated to provide an overall picture of the sample. Paired samples t-tests were conducted to compare general attitudes (regarding physical activity, aerobic, and strength exercises) towards the parents’ own exercise behaviour and their general attitude towards their child performing these behaviours. In the same way, differences between specific attitudes towards aerobic and strength attitudes were examined. For two variables (“General attitude about child’s strength exercises”, and “Is your child allowed to participate in exercises with the emphasis on strength exercises?”) Pearson’s correlations were reported for parental and child demographics, behaviours, general attitudes relating to general physical activity and aerobic exercises, specific attitudes and parental assent (whether the parents allow their child to engage in aerobic exercises) (for interested readers: Spearman’s Rho can be found in Additional file 1). Only significant variables, with a *p*-value < .001 (to correct for multiple testing), were added into two separate linear regression models. The outcome variable of the first model was general parental attitude regarding their child’s strength exercise behaviour. In the second model, parental assent (whether the parents allow their child to participate in strength exercises) was included as an outcome.

When the participant indicated that their child was not allowed to participate in aerobic or strength exercises, the most important reason(s) for that decision was (were) recorded. These (qualitative) data were divided into categories by two independent raters (GtH & ES). After this individual categorization process, definite categories were chosen by consensus. Frequencies and percentages of themes are reported.

## Results

Characteristics of the study population are depicted in Table 2. Surveys were completed by an approximately equal number of mothers (43.6 %) and fathers (56.4 %). The majority of the participants reported medium or higher levels of education, with 45.2 % indicating that they had completed intermediate/high general secondary education or intermediate vocational education, and 32.5 % indicating that they had a college degree or higher. Gender of the children was also equally divided (48.4 % girls). The mean age of the children was 13.4 (±1.0 *SD*) years. With regard to weight status, 60.9 % of the parents were overweight (40.8 %) or obese (20.1 %) and for children these percentages were 12.0 % and 9.7 %, respectively). In general, parents reported that their children were more physically active (*M*_*child*_ *=* 319.1 min per week (*SD =* 401.9)) compared to themselves (*M*_*parent*_ =168.5 min per week (*SD* = 272.4)) *t*(313) = −6.15, *p* < .001.

**Table 2:** Background characteristics of the sample (*N* = 314)^a^

	Parent	Child
	M (sd)	M (sd)
Gender (Female:Male)	137:177	152:162
Age in years *(SD)*	45.8 (4.7)	13.4 (1.0)
Education level		
Low (%)	70 (22.3)	-
Medium (%)	142 (45.2)	-
High (%)	102 (32.5)	-
BMI (z) (*SD*)^b^	26.65 (4.51)	−0.05 (1.32)
Underweight (%)	4 (1.3)	36 (12.0)
Normal-weight (%)	119 (37.9)	199 (66.3)
Overweight (%)	128 (40.8)	36 (12.0)
Obese (%)	63 (20.1)	29 (9.7)
Physical activity in min/week (SD)	168.5 (272.4)	319.1 (401.9)
Aerobic exercise (%)	74 (38.5)	56 (24.1)
Mostly aerobic (%)	70 (36.5)	102 (44.0)
Both aerobic and strength (%)	38 (19.8)	62 (26.7)
Mostly strength (%)	9 (4.7)	11 (4.7)
Strength exercises (%)	1 (0.5)	1 (0.4)
No physical activity (%)	122 (38.9)	82 (26.1)

*Note.* 12 children had missing values for BMI/weight status; 2 children had an unrealistic BMI z-score (< −5) and were removed as advised by the WHO [28]

Type of physical activity behaviour (ranging from aerobic exercise to strength exercise); percentages for type of exercise were calculated after removing the children that were not physically active (no sport); percentage from total sample was calculated for children that were not physically active (no sport)

^a^All values are *N*’s, unless otherwise indicated. ^b^ a BMI score was calculated for the parents; a BMI z-score was calculated for the youngsters

### Types of physical activity behaviour and reasons for not being physically active

The majority of the parents reported that when they are physically active it tends to be characterised by aerobic exercise more than strength training (see Table 2). Parents identified the exercise of their children as ‘mostly aerobic’ (44.0 %). From the total sample, 38.9 % of parents were not physically active (no sport participation), as compared to 26.1 % of the children. Sixty-one percent of the inactive children (50 out of 82) had inactive parents.

**Fig. 1:**
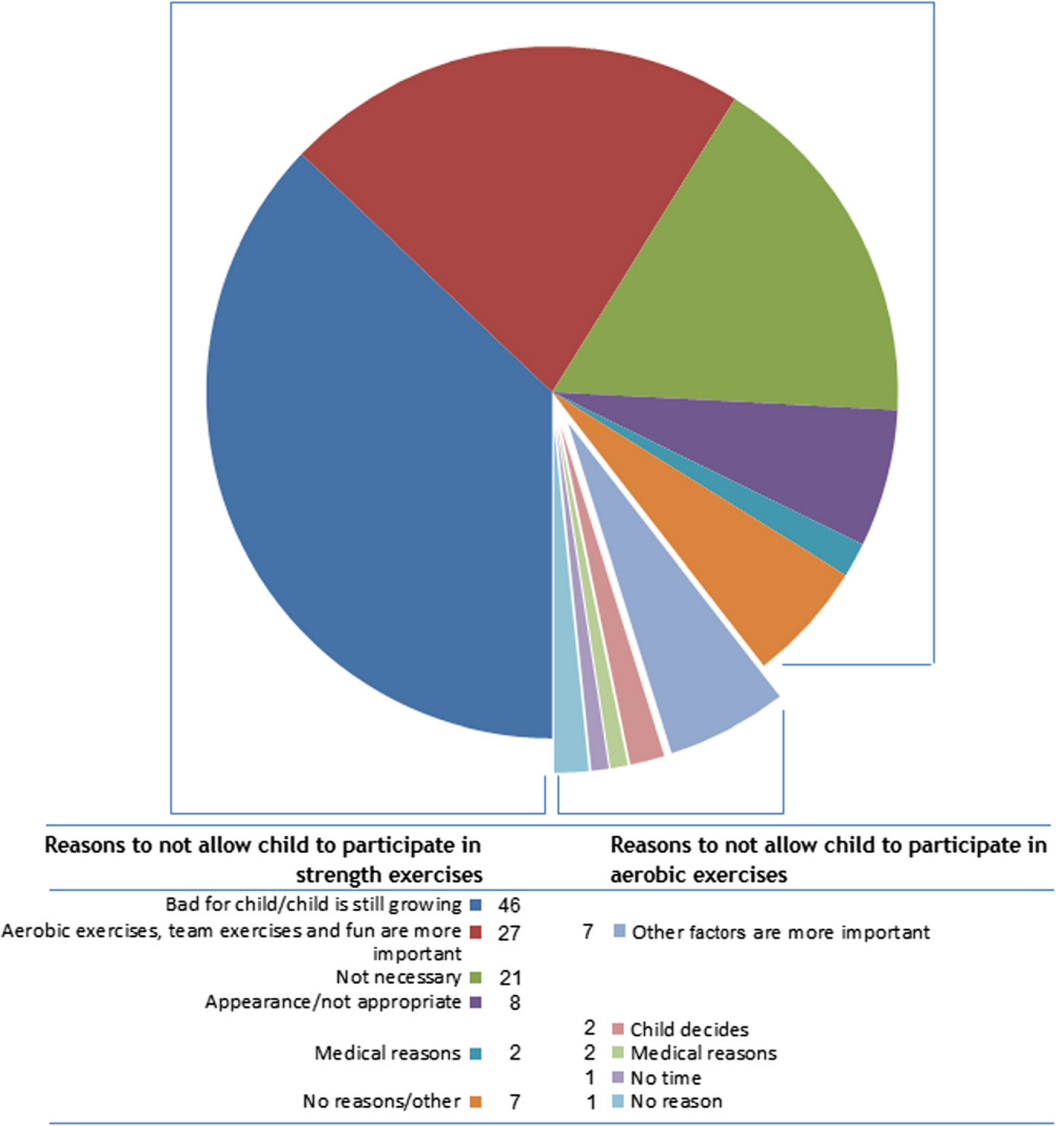
Parental reasons for not allowing their child to participate in exercises with an emphasis on aerobic and resistance components

We asked whether parents allowed their child to perform physical activity with the emphasis on aerobic exercises (*M* = 5.81, *SD* = 1.31; range 1–7) or on strength exercises (*M* = 4.32, *SD* = 1.67; range 1–7). These numbers indicate that most parents were positive about aerobic exercise, but less positive about their child participating in strength exercises. Thirteen parents (4.0 %) indicated that they preferred their child not to participate in exercises with an emphasis on aerobic components. The most important reason for this (mentioned by 7 out of 13 parents) was that they considered other factors (such as fun)to be more important; two parents indicated that their child decides, one mentioned time as reason and 1 mentioned medical reasons. Many more parents indicated that their child was not allowed to participate in exercises with an emphasis on strength (93 out of 314; 29.6 %). Parents reported that they worried that exercises with an emphasis on strength components were bad for their child’s health (*n* = 46; injuries and physical development). Additionally, parents believed that aerobic exercises, team exercises and fun were more important, (*n* = 27), or indicated that they thought strength exercises were not necessary for their child’s health (*n* = 21). Also, a small group (*n* = 8, of which 6 had daughters) thought that strength training was not appropriate for their child’s appearance (i.e., body builder image/activities that are seen as masculine). Other reasons mentioned included time*,* costs, and their child deciding for him/herself (see Fig. 1).

### Differences in attitudes of parent vs. child towards aerobic vs. strength exercises

As compared to their own general attitudes regarding types of physical activity behaviours (i.e., aerobic, strength, or a combination of both), parents reported more positive attitudes about their child’s physical activity in terms of general and aerobic exercise (see Table 3). No such difference was found with regard to strength exercises. Moreover, parental attitudes towards strength exercises were significantly more negative (all *p*’s < .001). Analyses for daughters and sons separately, or mothers and fathers, all showed comparable results (not reported; see Additional file 1).

**Table 3:** Differences in general parental attitudes with regard to their own and their child’s exercise behaviour (*N* = 314)

		Parent	Child	t (df)	*p*
		M (sd)	M (sd)		
General attitude (1–7)	Sport	5.33 (.96)	5.87 (.87)	−11.84 (313)	< .001
	Aerobic	5.18 (1.02)	5.54 (.94)	−6.78 (313)	< .001
	Strength	4.09 (1.10)	4.03 (1.33)	.83 (313)	.41

We conducted an independent samples *t*-test to see whether there were differences between the specific attitudes regarding aerobic and strength exercise. All parents had more positive scores for aerobic as compared to strength exercise (see Table 4). Again no differences in outcomes were found when analysing the data in gender sub-groups.

**Table 4:** Differences in specific parental attitudes with regard to aerobic and strength exercise behaviour of their child (*N* = 314)

Determinant (1–7)	Aerobic	Strength	t (df)	*p*
	M (sd)	M (sd)		
Possible	5.36 (1.53)	4.20 (1.82)	−11,76 (313)	< .001
Facilities	5.54 (1.37)	4.74 (1.66)	−9,87 (313)	< .001
Fit/strong	3.63 (1.63)	4.39 (1.55)	−8,35 (313)	< .001
Worse/better	4,58 (1.33)	4.15 (1.16)	−5,85 (313)	< .001
Enjoyable	4,42 (1.31)	3.61 (1.30)	−9,62 (313)	< .001
Good	4,73 (1.45)	4.26 (1.35)	−5,69 (313)	< .001
Allowed when wanted	5,82 (1.08)	4.82 (1.38)	−12,50 (313)	< .001
Encouraged when wanted	5,75 (1.02)	4.33 (1.46)	−16,96 (313)	< .001
Expectation	4,74 (1.62)	2.88 (1.54)	−18,35 (313)	< .001

*Note.* Please note that the specific questions belonging to the constructs above are described in Table 1

### Attitudinal correlates of strength exercise behaviour

Pearson’s correlations were reported for parental and child demographics, behaviours, general attitudes about general physical activity and aerobic exercises, specific attitudes and parental assent (whether parents allowed their child to engage in aerobic exercises). Only significant variables, with a *p*-value < .001 (to correct for multiple testing), were added into two separate linear regression models. The outcome variable of the first model was general parental attitude regarding their child’s strength exercise behaviour. In the second model, parental assent (whether parents allowed their child to engage in strength exercises) was included as an outcome. Pearson’s correlations and regressions are reported for parental and child demographics, physical activity behaviours, general and specific attitudes about physical activity, strength and aerobic exercises, and parental assent (whether they allowed their child to engage in aerobic exercises), see Table 5. We reported correlations and regressions in one table, because the betas are sensitive to intercorrelations among the predictors. We first describe correlations, then report linear regression model 1, and finally regression model 2. Parents’ general attitudes about their child’s strength exercises were neither positive nor negative (M = 4.03 on a scale from 1 to 7). Of the variance in general attitude regarding the child’s strength exercise behaviour, 60 % could be explained by the variables in our model. The highest correlation was with the parental assent (whether they allow their child to participate in strength exercises) (*r* = .68). Most variance was explained by general parental attitude towards strength exercises, β = .44 (*r* = .55), parental encouragement when their child wants to participate in strength exercises, β = .37 (*r* = .65), and whether the parents allow their child to participate in strength exercises when the child wants to, β = .05 (*r* = .52). In addition to these determinants, parental estimations of whether their child enjoys strength exercises has made a significant contribution to the regression β = .13 (*r* = .40).

**Table 5:** Determinants of parental attitude about their child’s strength exercise behaviour and parental assent (whether or not they allowed their child to engage in strength exercises

	General attitude about child’s strength exercises			Is your child allowed to participate in exercises with the emphasis on strength exercises?		
	(*N* = 314)					
				(*N* = 314)		
Determinant	*r*	*β*	CI (95 %)	*r*	*β*	CI (95 %)
Age parent	-.11	*-*		-.03	*-*	
Age child	-.01	-		.05	*-*	
Gender parent (1 = M)	.06	-		.02	*-*	
Gender child (1 = M)	-.02	-		.02	*-*	
BMI parent	-.03	-		-.002	-	
BMI z-score child	-.16*	-		-.10	-	
Parent exercise (minutes/week) (*n* = 192)	.13	-		.13	-	
Child exercise (minutes/week) (*n* = 232)	-.06	-		-.03	-	
Kind of exercise parent (*n* = 192)^a^	.25*	-		.21	-	
Kind of exercise child (*n* = 232)^a^	-.06	-		.02	-	
Parental assent strength exercises child	.68**	NA		-	-	
Parental assent aerobic exercises child	.26**	.02	-.08, .12	.42**	.21**	.10, .24
Parental Attitudes about physical activity						
Attitude about parent physical activity	.24**	-.07	-.27, .08	.12	-	
Attitude about child physical activity	.15*	-		.10	-	
Attitudes about strength exercises						
Attitude about parent strength exercises	.55**	.44**	.39, .64	.32**	.12*	.04, .33
Attitude about child strength exercises	-	-		.68**	NA	
Possible	.30**	.07	-.02, .11	.30**	.09	-.001, .16
Facilities	.14*	-		.16*	-	
Fit/strong	-.15 *	-		-.07	-	
Worse/better	.20**	-.07	-.21, .06	.19*	-	
Enjoyable	.40**	.13*	.02, .24	.31**	.12*	.02, .29
Good	.23**	-.01	-.13, .11	.20**	.07	-.11, .13
Allowed when wanted	.52**	.05	−08, .18	.55**	.19*	.06, .40
Encouraged when wanted	.65**	.37**	.20, .47	.60**	.35**	.22, .57
Expectation	.42**	.003	-.09, .09	.29**	-.06	-.18, .05
Attitudes about aerobic exercises						
Parental attitude about aerobic exercises	.24**	.005	-.17, .18	.12	-	
Child attitude about aerobic exercises	.21**	.12	.01, .32	.12	-	
Possible	-.07	-		.003	-	
Facilities	-.06	-		.04	-	
Fit/strong	.01	-		.04	-	
Worse/better	.10	-		.11	-	
Enjoyable	.10	-		-.004	-	
Good	.08	-		.06	-	
Allowed when wanted	.07	-		.19*	-	
Encouraged when wanted	.07	-		.15*	-	
Expectation	.06	-		-.003	-	
Adjusted R^2^		.60			.46	

*Note.* *p < .01; ** *p* < .001; only correlates with *p* < .001 were added in the linear regression model. ^a^Kind of exercise: parents own estimation of the kind of exercise *(1 aerobic – 5 strength);* NB: The variable “is your child allowed to participate in exercises with the emphasis on strength exercises?” was not included as a predictor for “General attitude about child’s strength exercises” and vice versa

Of the variance of the parental assent (whether the parents allow their child to engage in strength exercises), 46 % could be explained by the determinants. Most variance was explained by parental encouragement when their child wants to participate in strength exercises, β = .35 (*r* = .60), whether the parents allow their child to participate in strength exercises when the child wants to, β = .21 (*r* = .55), and whether the parents allow their child to participate in aerobic exercises, β = .21 (*r* = .42). Other significant contributions were parental attitudes towards their own strength exercise behaviour, β = .12 (*r* = .32), and whether the parents thought that their child enjoy strength exercises, β = .11 (*r* = .31). Sensitivity analyses, where we ran the same statistical tests using only the parents with complete data revealed no difference in results from the analyses including the imputed values (*r*^*2*^ *=* .62, and .47 respectively; data not shown).

We selected parents (*n* = 46) who indicated that they think strength exercises are bad for their child, *and* indicated that their child was not allowed to participate in strength exercises (M = 2.65). We examined whether these parents would allow their child to participate in strength exercises if their child wanted to. Mean scores indicated that under that condition parents were neutral about their child’s participation in strength exercises (M = 3.73), and less negative about their own encouragement (M = 3.02). However, they did not expect their child to enjoy strength exercises (M = 3.18).

## Discussion

### Parents’ attitudes about child physical exercise

The literature on parents’ attitudes about their child’s exercise is limited, but indicates that there is a positive relation between parental attitudes and exercise behavior of children [29, 30]. These studies do not distinguish between aerobic and strength exercises. Our study consistently shows that parents have a positive attitude towards aerobic exercises, but a less positive attitude regarding strength exercises. Interestingly, when parents were asked to outline the reasons why they are negative about their child doing strength exercises, most mentioned (incorrectly) that such exercises are bad for their child, and that aerobic exercises, team exercises and having fun are more important. Please note that we only asked parents who did not allow their child to participate in strength exercises about their reasons for non-participation. As parental attitude about child strength exercises was most highly correlated with parental assent (whether or not parents allowed their child to participate in strength exercises), most of the reasons for non-participation will be accounted for by those parents (who did not allow their child to participate in strength exercises). However, parents who do allow their child to participate in strength exercises may also consider reasons for non-participation. In future research these reasons should also be identified.

One limitation of our study is that besides asking questions about their own behaviour and attitudes, we also asked the parents to supply information about their child’s behaviour and attitudes. Parental estimations of their own and their child’s physical activity behaviour was not optimally measured in this study and, ideally, more objective measures of physical activity behavior should be used (see also [31]). However, we were more interested in the relationships between variables than their absolute values. Moreover, it is unknown whether the children would describe their own behaviour in a similar way or differently. However, the focus of this study was on parents and in particular parents’ attitudes regarding strength training in their children. Parents who were negative about allowing their child to participate in strength exercises reported that they would be less negative if their child wanted to participate in those exercises. These parents also indicated that they did not think their child would like such exercises. However, these parents may never have discussed strength exercises with their child. Parents may not relate strength exercises with improving health or with fun (see e.g., [16, 17]). For the parents who indicated that they did not want their child to participate in strength training, the most important reasons were related to *negative* consequences for their child’s health. The data also showed that their idea of strength training was that it does not involve aerobic components, team play, and fun.

### Implications for interventions

In conclusion, this study shows that parents are more positive about aerobic exercises as compared to strength exercises for their child. When developing interventions that encourage the use of strength exercises in youngsters, an important target group to focus on is the children’s parents. Parents have a crucial role to play in children’s physical activity-related behaviour [18, 19]. We suggest testing interventions to increase parents’ understanding of the advantages of and possibilities (e.g., facilities) for strength training and the benefits of strength exercises on their child’s health.

## Additional file

Additional file 1:
**Research material, data, and SPSS syntaxes.** (ZIP 153 kb)

## Abbreviations

BMI: Body Mass Index
