# Supplementary material for: Aerobic and strength exercises for youngsters aged 12 to 15: what do parents think?
Source: BMC Public Health. 2015 Sep 30;15:994. doi: 10.1186/s12889-015-2328-7 (PMC4589906; doi:10.1186/s12889-015-2328-7)
Supplement: Additional file 1: — Research material, data, and SPSS syntaxes. (ZIP 153 kb) [file 12889_2015_2328_MOESM1_ESM.zip › spearmansRHO.docx]

|  | General attitude about child’s strength exercises  (*N* = 314) | | Is your child allowed to participate in exercises with the emphasis on strength exercises  (*N* = 314) | |
| --- | --- | --- | --- | --- |
| Determinant | *Spearmans Rho* | *r* | *Spearmans Rho* | *r* |
| Age parent | -.12 | *-.11* | -.01 | *-.03* |
| Age child | *.01* | *-.01* | *.05* | *.05* |
| Gender parent (1=M) | *.05* | *.06* | *.02* | *.02* |
| Gender child (1=M) | *.01* | *-.02* | *.03* | *.02* |
| BMI parent | -.04 | -.03 | .01 | -.002 |
| BMI z-score child | -.17 | -.11 | -.08 | -.06 |
| Parent exercise (minutes/week) (*n* = 192) | -.17 | .13 | .03 | .13 |
| Child exercise (minutes/week) (*n* = 232) | -.01 | -.06 | -.02 | -.03 |
| Kind of exercise parent (*n* = 192)^a^ | -.12 | .25 | .01 | .21 |
| Kind of exercise child (*n* = 232)^a^ | -.01 | -.06 | .01 | .02 |
|  |  |  |  |  |
| Allowance strength exercises child | .70 | .68 | - | - |
| Allowance aerobic exercises child | .27 | .26 | .44 | .42 |
|  |  |  |  |  |
| *Parents Attitudes about physical activity* | |  |  |  |
| Attitude about parent physical activity | .23 | .24 | .13 | .12 |
| Attitude about child physical activity | .17 | .15 | .12 | .10 |
|  |  |  |  |  |
| *Attitudes about strength exercises* | .49 | .55 | .32 | .32 |
| Attitude about parent strength exercises | - | - | .70 | .68 |
| Attitude about child strength exercises | .29 | .30 | .29 | .30 |
| Possible | .12 | .14 | .15 | .16 |
| Facilities | -.16 | -.15 | -.06 | -.07 |
| Fit/strong | .20 | .20 | .19 | .19 |
| Worse/better | .36 | .40 | .28 | .31 |
| Enjoyable | .22 | .23 | .22 | .20 |
| Good | .48 | .52 | .56 | .55 |
| Allowed when wanted | .64 | .65 | .61 | .60 |
| Encourage when wanted | .44 | .52 | .29 | .29 |
| Expectation |  |  |  |  |
| *Attitudes about aerobic exercises* |  |  |  |  |
| Parent attitude aerobic exercises | .22 | .24 | .11 | .12 |
| Child attitude aerobic exercises | .21 | .21 | .13 | .12 |
| Possible | -.04 | -.07 | .03 | .003 |
| Facilities | -.01 | -.06 | .07 | .04 |
| Fit/strong | .02 | .01 | .04 | .04 |
| Worse/better | .07 | .10 | .09 | .11 |
| Enjoyable | .09 | .10 | .02 | -.004 |
| Good | .06 | .08 | .04 | .06 |
| Allowed when wanted | .07 | .07 | .19 | .19 |
| Encourage when wanted | .10 | .07 | .16 | .15 |
| Expectation | .05 | .06 | .02 | .003 |
|  |  |  |  |  |
