# Supplementary figures and images for: Aerobic and strength exercises for youngsters aged 12 to 15: what do parents think?
Source: BMC Public Health. 2015 Sep 30;15:994. doi: 10.1186/s12889-015-2328-7 (PMC4589906; doi:10.1186/s12889-015-2328-7)

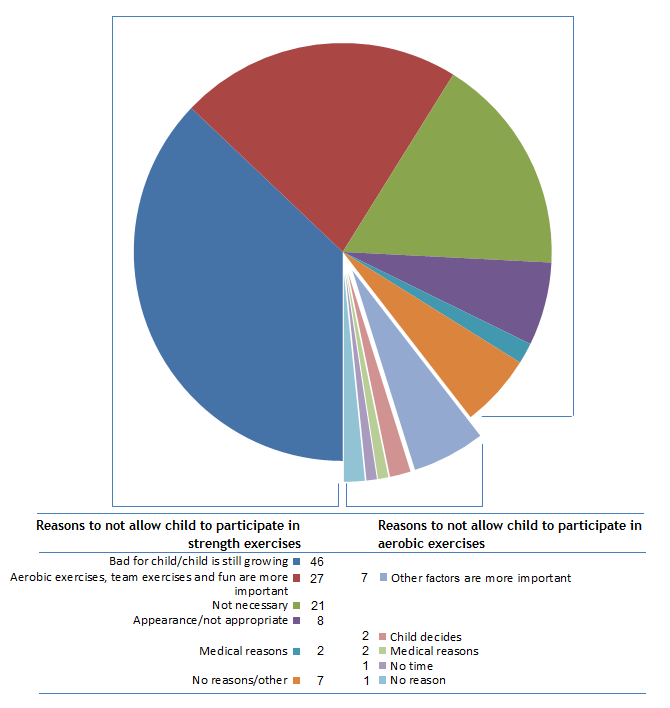

Supplement: Additional file 1: — Research material, data, and SPSS syntaxes. (ZIP 153 kb) [file 12889_2015_2328_MOESM1_ESM.zip › reasons2.png]
